# Supplementary material for: Newcastle Disease Genotype VII Prevalence in Poultry and Wild Birds in Egypt
Source: Viruses. 2022 Oct 13;14(10):2244. doi: 10.3390/v14102244 (PMC9607356; doi:10.3390/v14102244)
Supplement: Supplementary file 1 [file viruses-14-02244-s001.zip › viruses-1893405-supplementary.pdf]

Supplementary material

Supplementary Table S1. The genetic relatedness among the NDV strains. Phylogenetic analysis of a total of 130 NDV strains, including our five isolates (in bold), was performed based on the full F gene coding region. The analysis included 125 representative sequences that belong to different genotyping groups. Representative datasets were created by an international consortium of NDV experts for the needs of objective classification of NDV isolates.

	**NDV/Chicken/Egypt/ALEX/ZU-NM99/2019**	**NDV/Chicken/Egypt/ALEX/ZU-NM97/2019**	**NDV/Chicken/Egypt/NOR/ZU-NM76/2019**	**NDV/Black-crowned night heron/Egypt/POR/ZU-NM85/2019**	**NDV/Duck/Egypt/DAK/ZU-NM09/2019**
**NDV/Chicken/Egypt/ALEX/ZU-NM99/2019**		98.014	100	100	98.014
**NDV/Chicken/Egypt/ALEX/ZU-NM97/2019**	98.014		98.014	98.014	100
**NDV/Chicken/Egypt/NOR/ZU-NM76/2019**	100	98.014		100	98.014
**NDV/Black-crowned night heron/Egypt/POR/ZU-NM85/2019**	100	98.014	100		98.014
**NDV/Duck/Egypt/DAK/ZU-NM09/2019**	98.014	100	98.014	98.014	
XIV.2_XIV_b_HF969187_chicken_Nigeria_NIE08_453_2008	86.883	87.425	86.883	86.883	87.425
XIV.2_XIV_b_HF969210_chicken_Nigeria_NIE10_139_2011	86.763	87.365	86.763	86.763	87.365
XIV.2_XIV_b_KY171990_chicken_Nigeria_KD_TW_03T_N45_720_2009	86.883	87.485	86.883	86.883	87.485
XIV.1_XIV_a_JN872165_chicken_Niger_VIR_1377_7_2006	87.304	87.545	87.304	87.304	87.545
XIV.1_XIV_a_HF969205_turkey_Nigeria_NIE09_2071_2009	87.064	87.485	87.064	87.064	87.485
XIV.1_XIV_a_JQ039386_chicken_Nigeria_VRD08_36_2008	87.545	87.906	87.545	87.545	87.906
XVII_XVII_b_HF969194_chicken_Nigeria_NIE08_2199_2009	87.665	87.906	87.665	87.665	87.906
XVII_XVII_a_HF969176_chicken_Nigeria_NIE10_310_2011	87.786	87.605	87.786	87.786	87.605
XVII_XVII_a_HF969191_chicken_Nigeria_NIE08_2042_2009	87.786	87.365	87.786	87.786	87.365
XII.2_XII_b_MF278927_goose_China_FS_SS_292_2013	88.207	88.267	88.207	88.207	88.267
XII.2_XII_b_JN627504_goose_China_GD_12_2011	88.267	88.327	88.267	88.267	88.327
XII.2_XII_b_JN627507_goose_China_GD_1003_2010	88.568	88.508	88.568	88.568	88.508
XII.1_XII_a_KU594618_chicken_Peru_Arequipa_VFAR_81_2015	87.064	87.004	87.064	87.064	87.004
XII.1_XII_a_KU594615_chicken_Peru_Apurimac_50009_2005	88.448	88.147	88.448	88.448	88.147
XII.1_XII_a_KU594616_gamecock_Peru_Lurin_40871_2004	88.448	88.147	88.448	88.448	88.147

XVIII.1_XVIII_a_JX518885_chicken_Mali_ML57051T_2010	88.387	88.568	88.387	88.387	88.568
XVIII.1_XVIII_a_FJ772455_Mauritania_1532_14_2006	88.628	88.809	88.628	88.628	88.809
XVIII.1_XVIII_a_JF966389_guinea_fowl_Mali_ML038_2007	88.207	88.387	88.207	88.207	88.387
XVIII.2_XVIII_b_JX518886_chicken_Mali_ML57072T_2010	89.29	88.748	89.29	89.29	88.748
XVIII.2_XVIII_b_HF969218_chicken_Ivory_Coast_CIV08_42_2007	88.869	88.688	88.869	88.869	88.688
XVIII.2_XVIII_b_HG326600_village_weaver_Ivory_Coast_CIV08_32_2006	89.11	88.929	89.11	89.11	88.929
XIII.2.2_XIII_b_KM056349_chicken_India_ndv42_gopalpura_4_2013	87.304	87.124	87.304	87.304	87.124
XIII.1.1_XIII_a_JN942043_roller_Tanzania_47385_11_2010	87.485	87.605	87.485	87.485	87.605
XIII.1.1_XIII_a_MF409241_chicken_Zambia_Chiwoko_2015	88.508	88.688	88.508	88.508	88.688
XIII.1.1_XIII_a_JN942034_ostrich_South_Africa_45445_3_1995	89.651	89.591	89.651	89.651	89.591
XIII.1.2_XIII_a_JQ267584_chicken_Iran_EMM_2_2008	89.35	89.29	89.35	89.35	89.29
XIII.1.2_XIII_a_JQ267579_chicken_Iran_EMM_7_2011	89.23	89.29	89.23	89.23	89.29
XIII.1.2_XIII_a_JQ267585_chicken_Iran_EMM_1_2008	89.11	89.17	89.11	89.11	89.17
XIII.2.2_XIII_b_KT734767_chicken_India_Polashbari_2014	88.147	88.267	88.147	88.147	88.267
XIII.2.2_XIII_b_KX372707_chicken_India_Nagpur_3_2011	87.244	87.244	87.244	87.244	87.244
XIII.2.1_XIII_b_GU182323_chicken_Pakistan_SPVC_Karachi_43_2008	87.545	87.726	87.545	87.545	87.726
XIII.2.1_XIII_b_GU182331_chicken_Pakistan_SPVC_Karachi_33_2007	88.327	88.387	88.327	88.327	88.387
XIII.2.1_XIII_b_KF113338_chicken_Pakistan_University_Diagnostic_Lab_12_2010	88.207	88.387	88.207	88.207	88.387
VII.2_VII_h_MF622047_chicken_South_Africa_RBWW_3_2013	89.17	88.989	89.17	89.17	88.989
VII.2_VII_i_KU862293_Parakeet_Pakistan_Karachi_AW_1_2014	90.554	90.193	90.554	90.554	90.193
VII.2_VII_i_HQ697254_chicken_Indonesia_Banjarmasin_10_2010	90.854	90.493	90.854	90.854	90.493
VII.1.2_VII_f_DQ227246_goose_China_Jiangsu_JS02_1999	92.96	92.78	92.96	92.96	92.78
VII.1.2_VII_f_AY028995_fowl_China_A7_1996	93.081	92.9	93.081	93.081	92.9
VII.1.2_VII_f_GQ338309_pigeon_China_18_2003	93.562	93.381	93.562	93.562	93.381
VII.1.1_VII_l_KX268351_chicken_Iran_Behshahr_2015	93.682	93.201	93.682	93.682	93.201
VII.1.1_VII_j_KC542905_chicken_China_Liaoning_1_2009_2009	98.075	97.894	98.075	98.075	97.894
VII.1.1_VII_e_AB853927_chicken_Japan_Ibaraki_SG106_1999	94.344	93.923	94.344	94.344	93.923
VII.1.1_VII_b_EF589133_pheasant_China_98_Guizhou_1998	95.307	95.247	95.307	95.307	95.247
VII.1.1_VII_d_EF579733_chicken_China_Shandong_Pyan_2004	94.344	94.043	94.344	94.344	94.043
VII.2_VII_k_KY747479_chicken_Namibia_5620_2016	89.29	89.11	89.29	89.29	89.11
VII.2_VII_a_JN986837_chicken_Netherlands_152608_ancestral_1993	91.937	91.637	91.937	91.937	91.637
XI_XI_HQ266602_chicken_Madagascar_MG_725_2008	82.07	81.889	82.07	82.07	81.889
XI_XI_JX518882_chicken_Madagascar_MGMNJ_2009	81.408	81.227	81.408	81.408	81.227
XI_XI_JX518884_chicken_Madagascar_MGS1595T_2011	81.889	81.829	81.889	81.889	81.829
II_II_GU978777_chicken_USA_TX_GB_1948	82.912	83.032	82.912	82.912	83.032
II_II_AF077761_chicken_USA_Lasota_1946	82.912	83.032	82.912	82.912	83.032
II_II_JN872151_chicken_USA_Hitchner_B1_1947	83.032	83.153	83.032	83.032	83.153
XXI_VI_l_KC205479_chicken_Ethiopia_ETHMG1C_2011	85.68	85.319	85.68	85.68	85.319
X.1_X_a_FJ705468_mottled_duck_USA_TX_130_2011	84.271	83.848	84.271	84.271	83.848
X.1_X_a_KX857716_Redhead_USA_ndv42_AI09_4117_2009	84.055	83.634	84.055	84.055	83.634
X.2_X_b_FJ705466_mallard_99_376_1999	84.15	84.15	84.15	84.15	84.15
X.2_X_b_KX857721_Mallard_USA_MN_AI10_3434_2010	83.755	83.755	83.755	83.755	83.755

V.1_V_b_JN872194_chicken_Honduras_498109_15_2007	84.717	84.717	84.717	84.717	84.717
V.1_V_b_JN942027_fighting_cock_Nicaragua_95066_9_2001	85.499	85.86	85.499	85.499	85.86
V.1_V_b_JN872189_parrot_USA_Coast_8278_1982	86.763	86.763	86.763	86.763	86.763
V.2_V_c_JQ697744_chicken_Mexico_NC04_635_2010	87.417	87.477	87.417	87.417	87.477
V.2_V_c_EU518682_Dove_Mexico_Distrito_Federal_462_2004	87.665	87.605	87.665	87.665	87.605
V.2_V_c_EU518684_chicken_Mexico_Estado_de_Mexico_466_2006	87.365	87.425	87.365	87.365	87.425
XIX_V_a_FJ705456_cormorant_USA_MN_92_40140_1992	85.723	85.723	85.723	85.723	85.723
XIX_V_a_JN942024_cormorant_USA_WI_272409_2003	86.161	85.68	86.161	86.161	85.68
XIX_V_a_KC433530_cormorant_USA_FL_41105_2012	85.379	85.259	85.379	85.379	85.259
XXI.2_VI_i_KU377535_Turtle_dove_Italy_12VIR1876_1_2012	85.86	85.56	85.86	85.86	85.56
XXI.2_VI_i_JN638234_dove_Italy_11RS98_102VIR_2011	85.8	85.379	85.8	85.8	85.379
XXI.2_VI_i_KU377533_Turtle_dove_Italy_10VIR7155_2010	85.74	85.319	85.74	85.74	85.319
XVI_XVI_JX915242_chicken_Dominican_Republic_28138_4_1986	85.018	85.018	85.018	85.018	85.018
XVI_XVI_JX186997_chicken_Dominican_Republic_867_2008	83.694	84.055	83.694	83.694	84.055
I.2_I_b_KC503453_American_green_winged_teal_USA_AK_44493_716_2009	84.176	84.116	84.176	84.176	84.116
I.2_I_b_AY965079_duck_Russia_FarEast_2713_2001	84.717	84.777	84.717	84.717	84.777
I.2_I_b_HG326605_spur_winged_goose_Nigeria_NIE08_121_2008	84.416	84.416	84.416	84.416	84.416
I.1.1_I_a_M24693_chicken_Australia_Queensland_1966	85.078	84.958	85.078	85.078	84.958
I.1.1_I_a_AY935490_chicken_Australia_2_1334_2002	85.078	84.898	85.078	85.078	84.898
I.1.1_I_a_AY935495_chicken_Australia_99_868_hi_1999	85.439	85.199	85.439	85.439	85.199
I.1.2.2_I_d_KC503476_northern_pintail_USA_AK_44500_136_2009	84.116	83.875	84.116	84.116	83.875
I.1.2.2_I_d_AB465607_chicken_Japan_Ishi_1962	85.018	84.958	85.018	85.018	84.958
I.1.2.2_I_d_KC503479_redpoll_Russia_Nikita_530_FFNK2_2008	83.935	83.815	83.935	83.935	83.815
I.1.2.1_I_c_KX352834_gull_Russia_Tyva_14_2014	82.671	82.732	82.671	82.671	82.732
I.1.2.1_I_c_EF564816_redknot_USA_NJ_A_101_1383_2001	83.273	83.454	83.273	83.273	83.454
I.1.2.1_I_c_GQ918280_black_headed_gull_Sweden_1994	84.176	84.296	84.176	84.176	84.296
XXI.1.2_VI_m_KY042141_Pigeon_Pakistan_Jallo_Lahore_221B_2016	86.161	86.522	86.161	86.161	86.522
XXI.1.2_VI_m_KU862298_pigeon_Pakistan_Lahore_AW_2_2015	86.221	86.522	86.221	86.221	86.522
XXI.1.2_VI_m_KY042135_Pigeon_Pakistan_22A_2015	86.643	87.064	86.643	86.643	87.064
III_III_GU182327_chicken_Pakistan_SPVC_Karachi_1_1974	84.838	84.958	84.838	84.838	84.958
III_III_EF201805_avian_Mukteswar_1940	85.078	85.199	85.078	85.078	85.199
III_III_MH996904_pigeon_Bulgaria_Novo_Selo_1161_1995	85.018	85.138	85.018	85.018	85.138
XXI.1.1_VI_g_KY042136_Pigeon_Pakistan_Lahore_125_2015	88.267	88.267	88.267	88.267	88.267
XXI.1.1_VI_g_JF824032_pigeon_Russia_Vladimir_687_2005	88.207	87.906	88.207	88.207	87.906
XXI.1.1_VI_g_KY042132_Pigeon_Egypt_73_OP_G29_2015	88.026	87.846	88.026	88.026	87.846
XX_VI_c_KY042142_quail_Korea_88_M_1988	88.267	88.207	88.267	88.267	88.207
XX_VI_c_AB853928_chicken_Japan_Ibaraki_SM87_1987	88.387	88.327	88.387	88.387	88.327
XX_VI_c_AF458016_chicken_China_ZhJ_2_1986	88.573	88.513	88.573	88.573	88.513
VI.2.1.2_VI_h_HG424627_pigeon_Nigeria_NIE13_92_2013	87.545	87.605	87.545	87.545	87.605
VI.2.2.2_VI_e_FJ480825_pigeon_China_PG_JS_1_2005	87.545	87.665	87.545	87.545	87.665
VI.2.2.2_VI_e_JX244794_pigeon_China_100_2008	87.966	88.087	87.966	87.966	88.087
VI.2.2.2_VI_e_KJ607163_pigeon_China_LJS_1_2004	87.846	87.846	87.846	87.846	87.846

VI.1_VI_b_AF109885_domestic_fowl_Great_Britain_GB1168_1984	88.508	88.568	88.508	88.508	88.568
VI.1_VI_b_FJ410145_pigeon_USA_NY_1984	88.448	88.387	88.448	88.448	88.387
VI.1_VI_b_FJ865434_pigeon_China_S_1_2002	88.267	88.327	88.267	88.267	88.327
VI.2.2.1_VI_f_JN872180_waterfowl_USA_TX_209682_2002	87.665	87.846	87.665	87.665	87.846
VI.2.2.1_VI_f_JN872182_pigeon_USA_12339_1998	87.605	87.545	87.605	87.605	87.545
VI.2.2.1_VI_f_JX901312_pigeon_USA_101_2001	87.485	87.184	87.485	87.485	87.184
VI.2.1.2_VI_h_HG326604_pigeon_Nigeria_NIE09_1898_2009	87.665	87.485	87.665	87.665	87.485
VI.2.1.2_VI_h_JX518532_laughing_dove_Kenya_B2_Isiolo_2012	87.786	87.244	87.786	87.786	87.244
VI.2.1.1.1_VI_n_MG018211_ECDO_USA_TX_1185_kidney_26981_3_A_2015	86.522	86.402	86.522	86.522	86.402
VI.2.1.1.1_VI_a_JX901367_pigeon_USA_PA_810_2008	87.545	87.726	87.545	87.545	87.726
VI.2.1.1.1_VI_a_JX901351_pigeon_USA_NJ_721_2007	87.726	87.786	87.726	87.726	87.786
VI.2.1.1.2.2_VI_k_MG840654.1_pigeon_China_Ningxia_2068_2016	86.101	86.041	86.101	86.101	86.041
VI.2.1.1.2.2_VI_k_KT163262_pigeon_China_SH_167_2013	86.643	86.582	86.643	86.643	86.582
VI.2.1.1.2.2_VI_k_JX901124_pigeon_Belgium_11_09620_2011	86.883	86.823	86.883	86.883	86.823
VI.2.1.1.2.1_VI_j_JX094510_pigeon_China_sms12_2012	87.064	87.064	87.064	87.064	87.064
VI.2.1.1.2.1_VI_j_JX901110_pigeon_Belgium_248_1998	88.327	88.387	88.327	88.327	88.387
VI.2.1.1.2.1_VI_j_JX486553_pigeon_China_LHLJ_110813_2011	87.906	87.726	87.906	87.906	87.726
IX_IX_AF458009_chicken_China_FJ_1_1985	85.732	85.973	85.732	85.732	85.973
IX_IX_FJ436303_chicken_China_ZJ_1_1986	85.62	85.74	85.62	85.62	85.74
IX_IX_FJ436302_chicken_China_F48E8_1948	85.68	85.8	85.68	85.68	85.8
IV_IV_AY741404_Fowl_UK_Herts_1933	87.425	87.425	87.425	87.425	87.425
IV_IV_MH996900_pullet_Bulgaria_Plovdiv_1153_1959	86.703	86.342	86.703	86.703	86.342
VIII_VIII_FJ751918_chicken_China_QH1_1979	87.485	87.665	87.485	87.485	87.665
XVI_XVI_JX915243_chicken_Mexico_Queretaro_452_1947_1947	88.628	88.929	88.628	88.628	88.929
VIII_VIII_AY734534_chicken_Argentina_Trenque_Lauquen_1970	88.267	88.508	88.267	88.267	88.508
VIII_VIII_JX012096_Malaysia_AF2240_1960	88.748	88.929	88.748	88.748	88.929

Supplementary Table S2. The genetic relatedness among the NDV isolates sequenced in this study. Phylogenetic analysis was done based on full genome sequence. Sequence similarities are represented as pairwise percentages, coloured from white to green for genetic dissimilarity to similarity.

	NDV/Chicken/Egypt/ALEX/ZU-NM97/2019	NDV/Black-crowned night heron/Egypt/POR/ZU-NM85/2019	NDV/Duck/Egypt/DAK/ZU-NM09/2019	NDV/Chicken/Egypt/NOR/ZU-NM76/2019	NDV/Chicken/Egypt/ALEX/ZU-NM99/2019
NDV/Chicken/Egypt/ALEX/ZU-NM97/2019		94.147	97.035	96.85	97.096
NDV/Black-crowned night heron/Egypt/POR/ZU-NM85/2019	94.147		94.481	95.628	96.194
NDV/Duck/Egypt/DAK/ZU-NM09/2019	97.035	94.481		96.873	97.182
NDV/Chicken/Egypt/NOR/ZU-NM76/2019	96.85	95.628	96.873		98.627
NDV/Chicken/Egypt/ALEX/ZU-NM99/2019	97.096	96.194	97.182	98.627	

Supplementary Table S3. Genetic similarity of closely branched strains from phylogenetic analysis of the fusion (F) protein gene from **Supplementary Figure S3**. Pairwise comparisons shown as percentage similarity of the open-reading frame.

	Egypt_76_chicken_2019	Egypt_85_night_heron_2019	Egypt_9_domestic_duck_2019	Egypt_97_chicken_2019	Egypt_99_chicken_2019
KY510687.1_NDV/chicken/IS/2/2017	98.014	98.014	97.954	97.954	98.014
JQ015297.1_NDV/chicken/China/SDYT03/2011	98.014	98.014	97.954	97.954	98.014
KU365650.1_NDV-FU4-EGYPT-NLQP-2014	98.255	98.255	98.195	98.195	98.255
KU365653.1_NDV-FU5-EGYPT-NLQP-2014	97.834	97.834	97.894	97.894	97.834
KY075892.1_NDV/chicken/Egypt/Ismailia29/2016	98.315	98.315	99.458	99.458	98.315
KY075888.1_NDV/chicken/Egypt/El-Arish16/2016	98.616	98.616	98.436	98.436	98.616

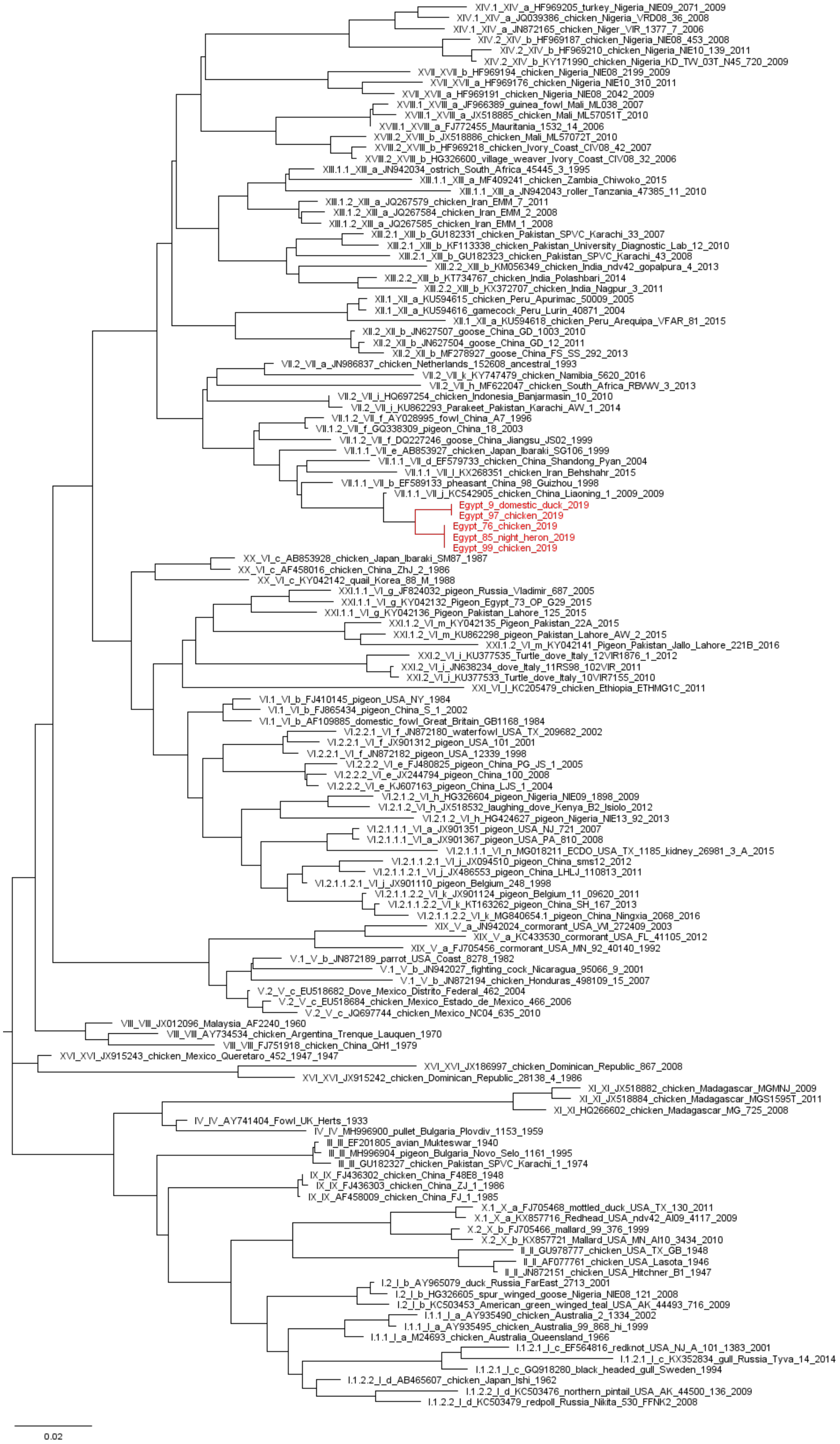
XIV.1_XIV_a_HF969205_turkey_Nigeria_NIE09_2071_2009
XIV.1_XIV_a_JQ039386_chicken_Nigeria_VRD08_36_2008
XIV.1_XIV_a_JN872165_chicken_Niger_VIR_1377_7_2006
XIV.2_XIV_b_HF969187_chicken_Nigeria_NIE08_453_2008
XIV.2_XIV_b_HF969210_chicken_Nigeria_NIE10_139_2011
XIV.2_XIV_b_KY171990_chicken_Nigeria_KD_TW_03T_N45_720_2009
XVII_XVII_b_HF969194_chicken_Nigeria_NIE08_2199_2009
XVII_XVII_a_HF969176_chicken_Nigeria_NIE10_310_2011
XVII_XVII_a_HF969191_chicken_Nigeria_NIE08_2042_2009
XVIII.1_XVIII_a_JF966389_guinea_fowl_Mali_ML038_2007
XVIII.1_XVIII_a_JX518885_chicken_Mali_ML57051T_2010
XVIII.1_XVIII_a_FJ772455_Mauritania_1532_14_2006
XVIII.2_XVIII_b_JX518886_chicken_Mali_ML57072T_2010
XVIII.2_XVIII_b_HF969218_chicken_Ivory_Coast_CIV08_42_2007
XVIII.2_XVIII_b_HG326600_village_weaver_Ivory_Coast_CIV08_32_2006
XIII.1.1_XIII_a_JN942034_ostrich_South_Africa_45445_3_1995
XIII.1.1_XIII_a_MF409241_chicken_Zambia_Chiwoko_2015
XIII.1.1_XIII_a_JN942043_roller_Tanzania_47385_11_2010
XIII.1.2_XIII_a_JQ267579_chicken_Iran_EMM_7_2011
XIII.1.2_XIII_a_JQ267584_chicken_Iran_EMM_2_2008
XIII.1.2_XIII_a_JQ267585_chicken_Iran_EMM_1_2008
XIII.2.1_XIII_b_GU182331_chicken_Pakistan_SPVC_Karachi_33_2007
XIII.2.1_XIII_b_KF113338_chicken_Pakistan_University_Diagnostic_Lab_12_2010
XIII.2.1_XIII_b_GU182323_chicken_Pakistan_SPVC_Karachi_43_2008
XIII.2.2_XIII_b_KM056349_chicken_India_ndv42_gopalpura_4_2013
XIII.2.2_XIII_b_KT734767_chicken_India_Polashbari_2014
XIII.2.2_XIII_b_KX372707_chicken_India_Nagpur_3_2011
XII.1_XII_a_KU594615_chicken_Peru_Apurimac_50009_2005
XII.1_XII_a_KU594616_gamecock_Peru_Lurin_40871_2004
XII.1_XII_a_KU594618_chicken_Peru_Arequipa_VFAR_81_2015
XII.2_XII_b_JN627507_goose_China_GD_1003_2010
XII.2_XII_b_JN627504_goose_China_GD_12_2011
XII.2_XII_b_MF278927_goose_China_FS_SS_292_2013
VII.2_VII_a_JN986837_chicken_Netherlands_152608_ancestral_1993
VII.2_VII_k_KY747479_chicken_Namibia_5620_2016
VII.2_VII_h_MF622047_chicken_South_Africa_RBVVW_3_2013
VII.2_VII_i_HQ697254_chicken_Indonesia_Banjarmasin_10_2010
VII.2_VII_i_KU862293_Parakeet_Pakistan_Karachi_AW_1_2014
VII.1.2_VII_f_AY028995_fowl_China_A7_1996
VII.1.2_VII_f_GQ338309_pigeon_China_18_2003
VII.1.2_VII_f_DQ227246_goose_China_Jiangsu_JS02_1999
VII.1.1_VII_e_AB853927_chicken_Japan_Ibaraki_SG106_1999
VII.1.1_VII_d_EF579733_chicken_China_Shandong_Pyan_2004
VII.1.1_VII_l_KX268351_chicken_Iran_Behshahr_2015
VII.1.1_VII_b_EF589133_pheasant_China_98_Guizhou_1998
VII.1.1_VII_j_KC542905_chicken_China_Liaoning_1_2009_2009
Egypt_9_domestic_duck_2019
Egypt_97_chicken_2019
Egypt_76_chicken_2019
Egypt_85_night_heron_2019
Egypt_99_chicken_2019
XX_VI_c_AB853928_chicken_Japan_Ibaraki_SM87_1987
XX_VI_c_AF458016_chicken_China_ZhJ_2_1986
XX_VI_c_KY042142_quail_Korea_88_M_1988
XXI.1.1_VI_g_JF824032_pigeon_Russia_Vladimir_687_2005
XXI.1.1_VI_g_KY042132_Pigeon_Egypt_73_OP_G29_2015
XXI.1.1_VI_g_KY042136_Pigeon_Pakistan_Lahore_125_2015
XXI.1.2_VI_m_KY042135_Pigeon_Pakistan_22A_2015
XXI.1.2_VI_m_KU862298_pigeon_Pakistan_Lahore_AW_2_2015
XXI.1.2_VI_m_KY042141_Pigeon_Pakistan_Jallo_Lahore_221B_2016
XXI.2_VI_i_KU377535_Turtle_dove_Italy_12VIR1876_1_2012
XXI.2_VI_i_JN638234_dove_Italy_11RS98_102VIR_2011
XXI.2_VI_i_KU377533_Turtle_dove_Italy_10VIR7155_2010
XXI_VI_l_KC205479_chicken_Ethiopia_ETHMG1C_2011
VI.1_VI_b_FJ410145_pigeon_USA_NY_1984
VI.1_VI_b_FJ865434_pigeon_China_S_1_2002
VI.1_VI_b_AF109885_domestic_fowl_Great_Britain_GB1168_1984
VI.2.2.1_VI_f_JN872180_waterfowl_USA_TX_209682_2002
VI.2.2.1_VI_f_JX901312_pigeon_USA_101_2001
VI.2.2.1_VI_f_JN872182_pigeon_USA_12339_1998
VI.2.2.2_VI_e_FJ480825_pigeon_China_PG_JS_1_2005
VI.2.2.2_VI_e_JX244794_pigeon_China_100_2008
VI.2.2.2_VI_e_KJ607163_pigeon_China_LJS_1_2004
VI.2.1.2_VI_h_HG326604_pigeon_Nigeria_NIE09_1898_2009
VI.2.1.2_VI_h_JX518532_laughing_dove_Kenya_B2_Isiolo_2012
VI.2.1.2_VI_h_HG424627_pigeon_Nigeria_NIE13_92_2013
VI.2.1.1.1_VI_a_JX901351_pigeon_USA_NJ_721_2007
VI.2.1.1.1_VI_a_JX901367_pigeon_USA_PA_810_2008
VI.2.1.1.1_VI_n_MG018211_ECDO_USA_TX_1185_kidney_26981_3_A_2015
VI.2.1.1.2.1_VI_j_JX094510_pigeon_China_sms12_2012
VI.2.1.1.2.1_VI_j_JX486553_pigeon_China_LHLJ_110813_2011
VI.2.1.1.2.1_VI_j_JX901110_pigeon_Belgium_248_1998
VI.2.1.1.2.2_VI_k_JX901124_pigeon_Belgium_11_09620_2011
VI.2.1.1.2.2_VI_k_KT163262_pigeon_China_SH_167_2013
VI.2.1.1.2.2_VI_k_MG840654.1_pigeon_China_Ningxia_2068_2016
XIX_V_a_JN942024_cormorant_USA_WI_272409_2003
XIX_V_a_KC433530_cormorant_USA_FL_41105_2012
XIX_V_a_FJ705456_cormorant_USA_MN_92_40140_1992
V.1_V_b_JN872189_parrot_USA_Coast_8278_1982
V.1_V_b_JN942027_fighting_cock_Nicaragua_95066_9_2001
V.1_V_b_JN872194_chicken_Honduras_498109_15_2007
V.2_V_c_EU518682_Dove_Mexico_Distrito_Federal_462_2004
V.2_V_c_EU518684_chicken_Mexico_Estado_de_Mexico_466_2006
V.2_V_c_JQ697744_chicken_Mexico_NC04_635_2010
VIII_VIII_JX012096_Malaysia_AF2240_1960
VIII_VIII_AY734534_chicken_Argentina_Trenque_Lauquen_1970
VIII_VIII_FJ751918_chicken_China_QH1_1979
XVI_XVI_JX915243_chicken_Mexico_Queretaro_452_1947_1947
XVI_XVI_JX186997_chicken_Dominican_Republic_867_2008
XVI_XVI_JX915242_chicken_Dominican_Republic_28138_4_1986
XI_XI_JX518882_chicken_Madagascar_MGMNJ_2009
XI_XI_JX518884_chicken_Madagascar_MGS1595T_2011
XI_XI_HQ266602_chicken_Madagascar_MG_725_2008
IV_IV_AY741404_Fowl_UK_Herts_1933
IV_IV_MH996900_pullet_Bulgaria_Plovdiv_1153_1959
III_III_EF201805_avian_Mukteswar_1940
III_III_MH996904_pigeon_Bulgaria_Novo_Selo_1161_1995
III_III_GU182327_chicken_Pakistan_SPVC_Karachi_1_1974
IX_IX_FJ436302_chicken_China_F48E8_1948
IX_IX_FJ436303_chicken_China_ZJ_1_1986
IX_IX_AF458009_chicken_China_FJ_1_1985
X.1_X_a_FJ705468_mottled_duck_USA_TX_130_2011
X.1_X_a_KX857716_Redhead_USA_ndv42_AI09_4117_2009
X.2_X_b_FJ705466_mallard_99_376_1999
X.2_X_b_KX857721_Mallard_USA_MN_AI10_3434_2010
II_II_GU978777_chicken_USA_TX_GB_1948
II_II_AF077761_chicken_USA_Lasota_1946
II_II_JN872151_chicken_USA_Hitchner_B1_1947
I.2_I_b_AY965079_duck_Russia_FarEast_2713_2001
I.2_I_b_HG326605_spur_winged_goose_Nigeria_NIE08_121_2008
I.2_I_b_KC503453_American_green_winged_teal_USA_AK_44493_716_2009
I.1.1_I_a_AY935490_chicken_Australia_2_1334_2002
I.1.1_I_a_AY935495_chicken_Australia_99_868_hi_1999
I.1.1_I_a_M24693_chicken_Australia_Queensland_1966
I.1.2.1_I_c_EF564816_redknot_USA_NJ_A_101_1383_2001
I.1.2.1_I_c_KX352834_gull_Russia_Tyva_14_2014
I.1.2.1_I_c_GQ918280_black_headed_gull_Sweden_1994
I.1.2.2_I_d_AB465607_chicken_Japan_Ishi_1962
I.1.2.2_I_d_KC503476_northern_pintail_USA_AK_44500_136_2009
I.1.2.2_I_d_KC503479_redpoll_Russia_Nikita_530_FFNK2_2008
0.02

Supplementary Figure S1. A maximum-likelihood phylogenetic tree of the full fusion, F gene sequences of members of the Class II Newcastle disease virus strains. The analysis involved 130 nucleotide sequences created by an international consortium of NDV experts for the needs of objective classification of NDV isolates (Dymitrov et al, 2019). Five samples sequenced in this study (red) clustered with China, Liaoning 2009, a group VII strain. GTR nucleotide substitution model, with among-site rate variation modelled using a discrete gamma distribution was performed to generate evolutionary history. Evolutionary analyses were conducted in MEGA11.

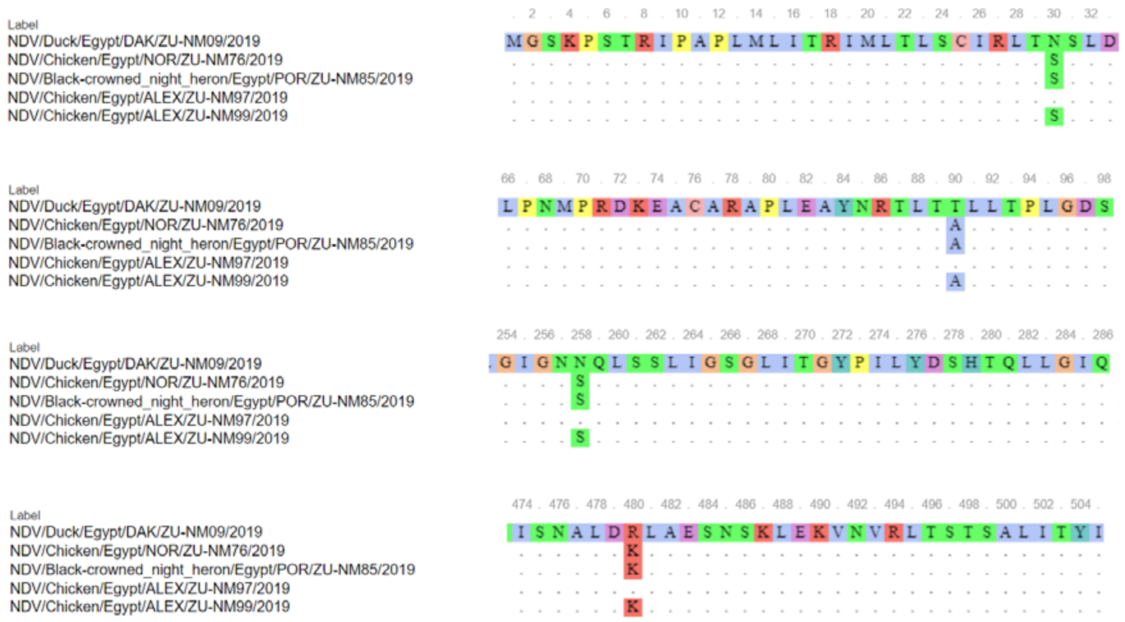

Supplementary Figure S2. Multiple sequence alignment of fusion protein (F) of Newcastle disease virus (NDV) strains sequenced in this study. Differences in amino acid compositions between the NDV strains are highlighted with position number listed above. Amino acids are

coloured by default using RasMol 'amino' colour scheme according to traditional amino acid properties. Identical amino acids are indicated by a (.).

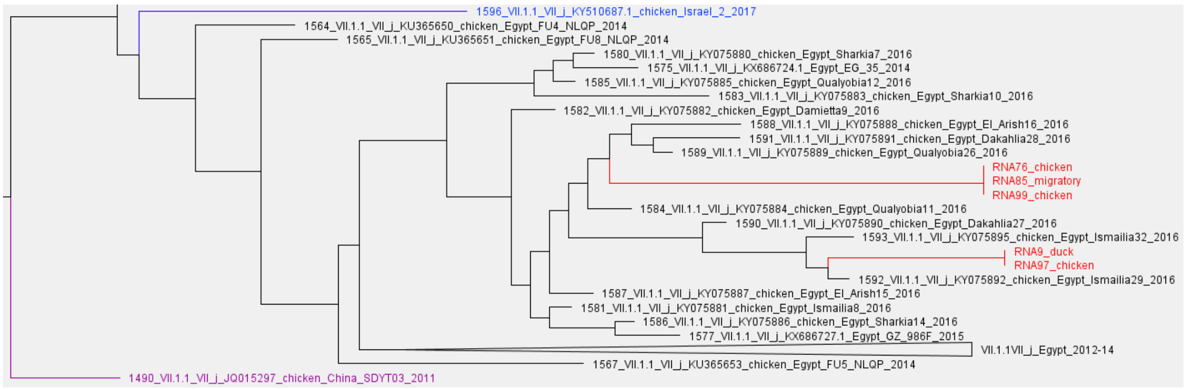

Supplementary Figure S3. Phylogenetic tree of the nucleotide sequences of the fusion (F) protein gene of Newcastle disease virus strains representing the genotype VII subgroup viruses (*n=559*). The evolutionary history was generated using GTR nucleotide substitution model, with among-site rate variation modelled using a discrete gamma distribution and 500 bootstrap replicate tests. The analysis involved 559 nucleotide sequences, including five F gene sequences in this study (red). Closely related strains outside of the Egyptian strains are an Israeli strain (blue) and a Chinese strain (purple). Visualised and edited in FigTree.
